# Light-sheet microscopy imaging of a whole cleared rat brain with Thy1-GFP transgene

**DOI:** 10.1038/srep28209

**Published:** 2016-06-17

**Authors:** Marzena Stefaniuk, Emilio J. Gualda, Monika Pawlowska, Diana Legutko, Paweł Matryba, Paulina Koza, Witold Konopka, Dorota Owczarek, Marcin Wawrzyniak, Pablo Loza-Alvarez, Leszek Kaczmarek

**Affiliations:** 1 Nencki Institute, Pasteura 3, 02-093 Warsaw, Poland; 2 Institut de Ciencies Fotoniques (ICFO), Barcelona Institute of Science and Technology, 08860, Castelldefels (Barcelona), Spain

## Abstract

Whole-brain imaging with light-sheet fluorescence microscopy and optically cleared tissue is a new, rapidly developing research field. Whereas successful attempts to clear and image mouse brain have been reported, a similar result for rats has proven difficult to achieve. Herein, we report on creating novel transgenic rat harboring fluorescent reporter GFP under control of neuronal gene promoter. We then present data on clearing the rat brain, showing that FluoClearBABB was found superior over passive CLARITY and CUBIC methods. Finally, we demonstrate efficient imaging of the rat brain using light-sheet fluorescence microscopy.

Mapping neural circuits that often span distant areas of the brain requires imaging of the entire brain at cellular resolution. However, light scattering in the brain tissue limits the depth of optical imaging. Therefore, until recently, whole-brain imaging was mostly achieved by the slow and labor-intensive method of sectioning and reconstruction^1^. Recent advances have provided an alternative approach. Optical clearing is a procedure that renders the brain transparent to light by removing the main scattering source – refractive index (RI) differences within the tissue. Since the potential of chemical clearing for imaging of intact brains was first demonstrated^2^, several other clearing methods yielding better transparency and lower fluorescence quenching have been developed^3^,^4^,^5^,^6^. However, although numerous improvements and modifications have been reported since then^7^,^8^,^9^,^10^,^11^, whole brain clearing still remains challenging, especially for adult, well-myelinated tissue^9^,^12^,^13^.

Another important advancement in brain imaging was applying light-sheet fluorescence microscopy (LSFM) to cleared brains^2^,^14^,^15^,^16^. By scanning the sample volume plane-by-plane instead of point-by-point, LSFM allows fast imaging of large specimens with sufficient resolution for quantitative neuroanatomy^17^,^18^.

The brain clearing procedures have originally been developed and optimized for mouse brain. Processing rat brains is more challenging, not only because of their size, but also higher degree of myelination. Despite the wide use of this species in neuroscience and its superiority in some experiments, including behavioral and physiological investigations, whole-brain clearing and subsequent imaging of an adult rat brain has not been, to our knowledge, reported, although scalability of clearing of periadolscent to adult rat brain has been described^19^.

In this work we present whole brain imaging of adult rat brain that has been cleared using the FluoClearBABB method, based on dehydration in increasing concentrations of tert-butanol^10^. For this, transgenic rat expressing GFP under Thy1 promoter, created by us, is used. FluoClearBABB is a simple, inexpensive clearing protocol yielding robust samples that can be easily imaged and stored in the clearing solution. We found that, of the protocols that we tested, that is PACT (passive CLARITY technique), CUBIC and FluoClearBABB, the latter is superior in preserving GFP fluorescence and at the same time providing sufficient tissue transparency for whole-rat brain lightsheet microscopy imaging.

## Results

### Production of Thy1-GFP transgenic rats

Transgenic Thy1-GFP Wistar rats were obtained by conventional pronuclear microinjection method. The successful transgenesis was confirmed by PCR analysis of samples derived from transgenic animals. All experiments were performed on heterozygous animals.

### Confocal microscopy imaging of the Thy1-GFP transgenic rat brain

**Figure 1:**
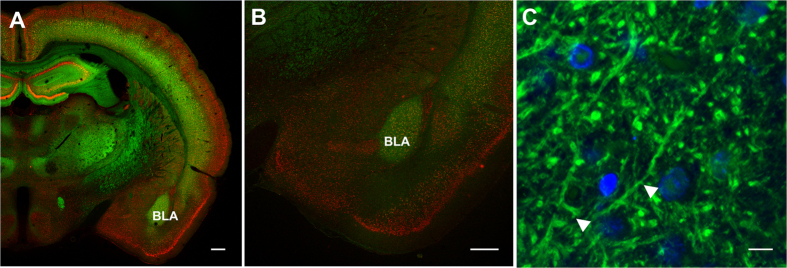
Confocal microscopy images of immunostained GFP expression in the Thy1-GFP transgenic rat brain. (**A**) Half brain coronal section; EC Plan-Neofluar 10x/0.30, tile images, maximum projection from Z-stack, scale bar: 500 μm; (**B**) Magnified image of the area with basolateral amygdala (BLA); EC Plan-Neofluar 10x/0.30, tile images, maximum projection from Z-stack, scale bar: 500 μm; (**C**) High resolution image of the basolateral amygdala, note dendritic spines (white arrows); Plan-Apochromat 40x/1.4 Oil DIC, maximum projection from Z-stack, scale bar 5 μm. Immunostaining: anti-GFP (green); anti-NeuN for neurons (red), DAPI for nuclei (blue).

To verify the GFP transgene expression and analyze its pattern, the rat brains were cut into sections and stained with antibodies. Anti-GFP staining (used here to enhance the GFP signal) revealed that the cortical layers, subcortical regions such as basal amygdala, and the hippocampus expressed the highest levels of the transgene, whereas low expression was noted in thalamus and central amygdala. No expression could be observed in hypothalamic nuclei. Figure 1A shows coronal brain section stained with anti-GFP and anti-NeuN (neuronal cell body marker) antibodies. Strong anti-GFP staining in the hippocampus, cerebral cortex, ventrolateral and ventromedial thalamic nuclei and basolateral amygdala is visible. Figure 1B presents a magnified view of the area encompassing basolateral amygdala, and Fig. 1C shows a high resolution image of this region, in this case stained with anti-GFP as well as DAPI to visualize cell nuclei. This panel demonstrates that dendrites and dendritic spines are strongly labeled with GFP.

### Development of light-sheet fluorescence microscopes for whole-brain imaging

Because of the lack of commercial microscopes that would meet our requirements to visualize the rat brain, we have built two light-sheet fluorescence microscopes, one in the Nencki Institute in Warsaw and one in ICFO in Castelldefels. The former setup was designed with maximum simplicity and cost-effectiveness in mind. It is based on the OpenSPIM configuration^20^ with modifications to account for much larger size of the brain samples and higher RI of mounting media used. It has single-sided illumination and single-sided detection with a 4X magnification detection objective. Combined with a 4 Megapixel camera, this corresponds to a 3.2 × 3.2 mm field of view and 1.57 μm per pixel resolution, which is sufficient for imaging cell bodies and dendrites.

The dual-illumination-dual-view (DIDV) setup in ICFO is more sophisticated and includes double-side illumination, realized with two identical illumination arms, as well as double-sided detection with two objectives with different magnification and numerical apertures. Double-sided illumination allows the light to penetrate from both sides to compensate for the residual absorption of the cleared sample. The two sides are illuminated and imaged sequentially, by blocking one of the illumination arms with a shutter. Furthermore, the system also includes two detection objectives. On one side we used a conventional photography macro objective, obtaining an extended field of view of 4.97 × 3.79 mm throughout the whole brain. Although this configuration simplifies the acquisition of large brain volumes at once, pixel size of 3.7 μm may be not enough when fine structures must be resolved. For that reason we have included a microscope objective on the other side of the chamber. This allows us to obtain higher resolution images of 1.05 μm per pixel, over a reduced field of view (1.46 × 1.09 mm). With this setup it is possible to obtain an overall picture of the whole brain using the macro photography objective and then, after rotating the sample, obtain a more relevant picture with increased resolution of the selected area. Both microscopes contain an XYZ translation stage that enables automated acquisition of adjacent image stacks. The setups are described in detail in Material and Methods section and shown in Supplementary Figure 1.

### Brain clearing

For clearing of the adult rat brain, we have tested three approaches, namely the passive CLARITY technique (PACT)^19^, CUBIC^6^ and FluoClearBABB^10^. Each of these approaches represents a different class of tissue-clearing methods: hydrogel embedding, hyperhydration and solvent-based dehydration, respectively. All these methods have previously been shown to work on entire mouse brains and to preserve fluorescence of various fluorescent proteins, including GFP.

**Figure 2:**
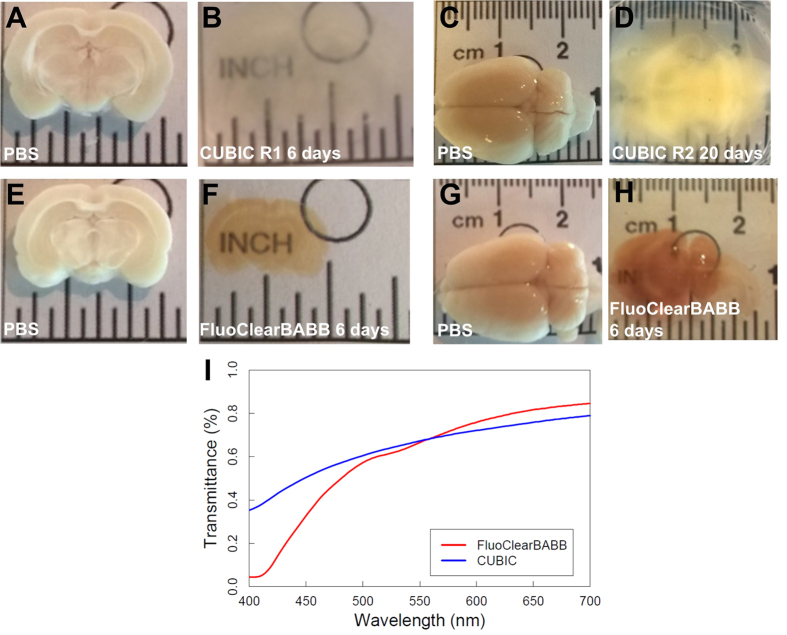
(**A–D**) Images of entire rat brains and 3 mm brain slices cleared according to the CUBIC protocol before and after incubation in CUBIC Reagent 1 and in Reagent 2 (R2). (**E–H**) Images of entire rat brains and 3 mm brain slices cleared using FluoClearBABB protocol before and after dehydration and incubation in BABB clearing solution. Note tissue swelling after CUBIC and shrinkage after FluoClearBABB. (**I**) Transmittance of a 3 mm brain slice cleared with CUBIC (blue) or FluoClearBABB (red).

To check the influence of sample size on clearing effectiveness, we performed clearing of thick (3 mm) brain slices and whole brains of adult rats. Macroscopic comparison (Fig. 2A,B for CUBIC and E,F for FluoClearBABB) revealed that for the brain slices both CUBIC and FluoClearBABB yielded good overall transparency. To confirm this, we measured the transmittance of the cleared slices. The result is shown in Fig. 2I for one CUBIC-cleared and one FluoClearBABB-cleared slice. Both slices show good transparency in the 500 to 700 nm region, with FluoClearBABB being slightly better for red light. However, as can be anticipated from the orange hue of the FluoClearBABB samples, their transmission drops dramatically for wavelengths shorter than 500 nm. It has to be noted that this measurement was made using a spectrophotometer designated for isotropic liquid samples, so the result represents averaged transmission of the entire illuminated region, neglecting transmission differences between different brain structures.

In the case of the whole brain, it was challenging to obtain sufficient level of transparency to reveal deep brain structures. We were able to meet this demand only in the case of FluoClearBABB (Fig. 2H). In the other brains, we were able to clear the cerebral cortex, but deeper structures, such as amygdala, remained inaccessible for imaging. We also attempted to extend the clearing time to compensate for the sample size. However, after 2 weeks of clearing in CUBIC Reagent 1 and further 1 week clearing in Reagent 2, the GFP fluorescence was extinguished and while transparency increased, it was still not sufficient for obtaining good quality images of, e.g., the hippocampus. In the case of prolonged PACT clearing, we also observed loss of fluorescence as well as a marked tissue swelling that resulted in non-uniform refractive index and strong distortion of image (not shown).

### Imaging of cleared brain slices

**Figure 3:**
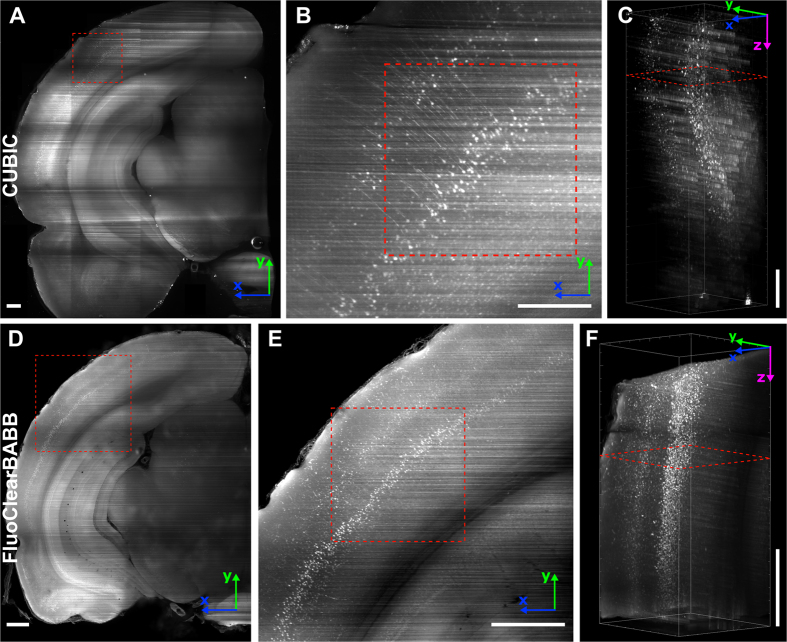
Light-sheet microscope imaging (4X, 0.1 NA objective) of rat brain slices cleared according to CUBIC (**A–C**) and FluoClearBABB **(D–F**) protocol. Both slices were initially the same size and thickness, the differences in size are a result of tissue expansion or shrinkage during clearing. To enable a comparison of clearing effectiveness, no background or noise removal was performed on these images. (**A**) Maximum projection of a 100 μm high stack at 800 μm depth from the sample surface. Image composed of 4 × 5 tiles. (**B**) Cortical neurons in the region of interest marked with a red square in A. (**C**) 3D rendering of the entire slice thickness corresponding to the region marked with a red square in B. The red square in C marks the depth at which the image shown in B was taken. (**D**) Maximum projection of a 100 μm-high stack at 800 μm depth from the sample surface. Image composed of 2 × 3 tiles. (**E**) Cortical neurons in the region of interest marked with a red square in D. (**F**) 3D rendering of the entire slice thickness corresponding to the region marked with a red square in E. The red square in F marks the depth at which the image shown in E was taken. Note that cell bodies are visible through the entire stack. Scale bar: 500 μm. x, y and z-axes are marked with blue, green and magenta arrows, respectively.

The light-sheet microscope images characterizing transparency and fluorescence preservation in CUBIC- and FluoClearBABB-cleared tissue are shown in Fig. 3. Figure 3A,D show examples of coronal sections about 800 μm from the sample surface for slices cleared with CUBIC and FluoClearBABB, respectively. In uncleared tissue imaging at such a depth is challenging even if two-photon microscopy is used. As shown in Fig. 3B,E, in both samples the cortical neurons are clearly visible. Finally, in Fig. 3C,F renderings of the entire slice thickness are shown. It can be seen that FluoClearBABB enables imaging of neurons in the entire thickness of the initially 3 mm thick slice, but in the CUBIC-cleared slice only about ¾ of the thickness can be imaged and more artefacts caused by scattering are visible. These results prove the superiority of FluoClearBABB for adult rat brain clearing.

**Figure 4:**
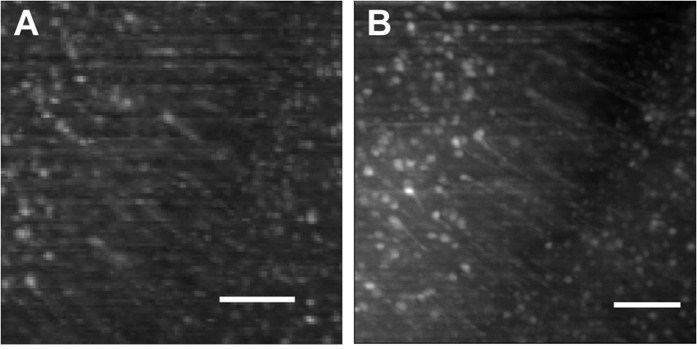
Cerebral cortex neurons of brain samples cleared with FluoClearBABB. Comparison of the resolution achieved with **(A)** macro photography objective and **(B)** 10x NA 0.45 microscope objective. The maximum projection of 10 images (3 μm appart) shows not only the nuclear body but also the axonal terminations of piramidal neurons. Scale bar: 100 μm.

It should also be noted that both PACT and CUBIC procedures resulted in an expansion of the brains, whereas FluoClearBABB method produced shrinkage of the tissue (compare Fig. 2D for CUBIC and 2H for FluoClearBABB). This effect influences image resolution, as illustrated by Fig. 3B,E. However, as shown in Fig. 4, the higher resolution objective available in the DIDV setup allows to see the neuronal morphology even for cells shrunk after FluoClearBABB clearing.

### Whole-brain imaging of FluoClearBABB-cleared rat brain

**Figure 5:**
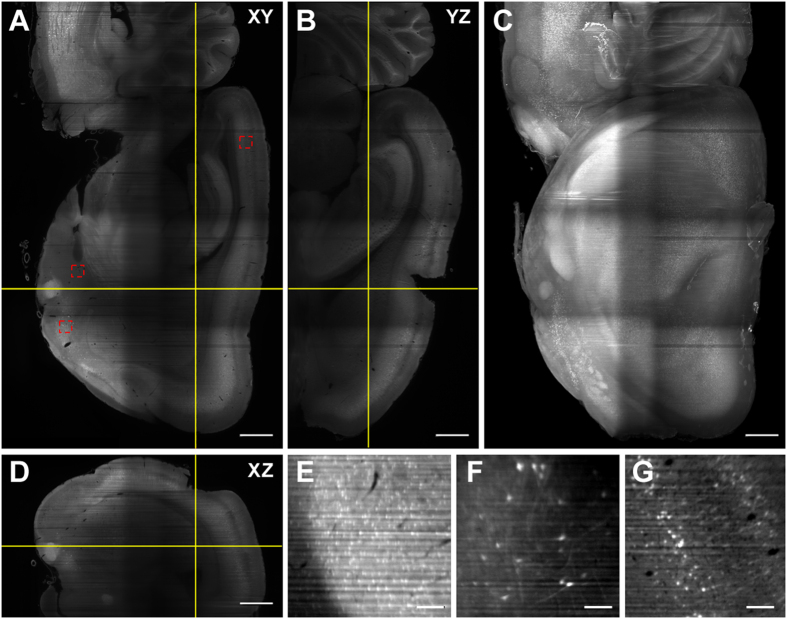
Light-sheet microscope imaging of a Thy1-GFP rat brain hemisphere cleared using FluoClearBABB technique. Orthogonal views: (**A**) A selected transverse plane at 3.44 mm from the top surface; (**B**) sagital view and (**D**) coronal view of the plain indicated in **(A)**. The data set was acquired using a macro objective and consists on the stitching of eight 3D stacks with double side illumination (four from each side). The maximum projection of the reconstructed dataset is displayed in (**C**). Scale bar: 1 mm. Three different regions of interest containing fluorescent neurons, highlighted in (**A**), are zoomed in (**E**) amygdala region at 2.22 mm; (**F**) subcortical neurons at 3.57 mm; and (**G**) cortical neurons at 2.11 mm. Scale bar: 100 μm.

To demonstrate that FluoClearBABB clearing was sufficient for whole-brain imaging, we cleared and imaged a hemisphere of a Thy1-GFP rat brain. The results are shown in Fig. 5. The 3D stack representing the entire hemisphere was obtained by stitching eight 3D stacks: four stacks obtained with left-side illumination and the other four with right-side illumination. Figure 5A shows a selected transverse plane at 3.44 mm from the surface. Two further planes showing a sagittal and a coronal view of the same brain, marked by solid lines in Fig. 5A, are shown in Fig. 5B,D, respectively. Note that these two images are orthogonal projections reconstructed from the original 3D stack, acquired transversally. A maximum projection is shown in Fig. 5C. Furthermore, the entire dataset is included in Supplementary Video 1 and 2. To illustrate the capability of imaging at cellular resolution, three selected regions, marked by squares in Fig. 5A, are shown in Fig. 5E–G.

The reconstructed orthogonal projections shown in Fig. 5B,D highlight an additional advantage of light-sheet microscopy. In contrast with confocal microscopy, the axial resolution of LSFM does not depend on the imaging objective, but on the light-sheet thickness which in turn depends on the lateral resolution of illumination objective. This makes it possible to obtain a good axial resolution even with long working distance, low-NA objectives^21^.

## Discussion

Whole-brain imaging with light-sheet fluorescent microscopy and optically cleared tissue and is a new, rapidly developing research field. In the last few years, a number of methods capable of clearing entire mouse brains have been demonstrated. However, we have found that not all these methods are directly transferable to the larger and more myelinated brain of the rat, which is the animal of choice for many neurobiological experiments. Herein, we demonstrate clearing and imaging of a rat brain hemisphere using the recently published FluoClearBABB method^10^. FluoClearBABB is a modified and refined version of the original BABB clearing protocol based on tissue dehydration with alcohol. The main advantages of this method are its simplicity, low cost and accessibility of reagents, as well as robustness of the cleared sample. While CUBIC or CLARITY-cleared samples need to be transferred to PBS for storage and then again into the refractive-index matching liquid for repeated imaging, BABB can be also used for long-term storage at 4 °C without loss of fluorescence.

Two other methods, CUBIC and PACT were tested in their simplest version, based on immersion in regularly exchanged clearing reagents. However, we have found that this approach is not suitable for entire rat brains or rat brain hemispheres, because long clearing time leads to fluorescence loss and sample degradation, possibly caused by the tissue swelling. A possible remedy would be to try active clearing, either by electrophoresis or by continuous, prolonged perfusion with clearing reagent^19^,^22^. However, these procedures are likely to be more challenging for rat than for mouse due to the larger size of the brain.

Furthermore, we demonstrate two home-built light-sheet microscope setups for brain imaging that use a stationary light sheet and standard air objectives. Although a simple configuration with one illumination objective and one detection objective is sufficient for imaging large samples with cellular resolution, better results are achieved by adding dual illumination to compensate for the absorption of the cleared brain and dual view using objectives with two different magnifications and fields of view (FOV) to rapidly find the region of interest within the whole brain before imaging under high-resolution conditions. The microscopes we describe represent simple and cost-effective, and therefore highly accessible, instruments which can be easily upgraded to work with scanning mirrors and specialized immersion objectives^7^,^16^, or even more complex illumination schemes, such as Bessel beams illumination^23^ that would allow extended FOV. As such, they can be seen as a valuable tool for rapid, whole-brain imaging, best fitted for tasks like tracing long-range neuronal projections^10^,^15^.

## Methods

### Generation of Thy1-GFP Transgenic Rats

In order to generate transgenic rats expressing GFP in neurons, we used the well-characterized Thy1 promoter^24^,^25^. The sequence for GFP was cloned into an XhoI site, incorporated previously in a way that the transgene replaces the exon 3 and its flanking introns^24^. Linear DNA fragment containing Thy1 cassette with GFP reporter was obtained by restriction enzymes digestion with EcoRI and PvuI. Approximately 7.5kb DNA fragments were excised from agarose gels and purified with Qiaex II kit (Qiagen). Eluted with TE embryo (10 mM Tris-HCl, 0.1 mM EDTA), filtered (0.22 μm, Millipore) and resuspended to the final concentration of 2 ng/μl, DNA solution was subsequently used for transgenesis. The fertilized embryos were obtained from superovulated Wistar rats and the standard pronuclear microinjection was performed. The embryos were then transferred to the foster mothers, as described previously^26^,^27^. Genotypes of obtained offspring were determined by PCR using the following primers: Thy1_F (TCTGAGTGGCAAAGGACCTTAGG) and GFP_R (CACGAACTCCAGCAGGACCATG). To this purpose, genomic DNA was isolated from ear fragments with DNeasy Tissue kit (Qiagen). PCR reactions were performed using Taq PCR Core Kit (Qiagen) and standard conditions were applied. Transgenic founders were crossed to Wistar wild type rats. All experiments were performed on heterozygous animals.

### Animals and Brain Isolation

Altogether, 15 transgenic rats were used for brain imaging. 2 brains were used for antibody staining and visualized with confocal microscope, 5 brains were subjected to PACT procedure, 4 to CUBIC and 4 to FluoroClearBABB. All procedures with the animals were performed in accordance with Polish law and EU directive (Directive 2010/63/EU) and approved by the First Warsaw Local Ethics Committee for Animal Experimentation located at the Nencki Institute. For brain isolation, rats were deeply anaesthetized with intraperitoneal injection of lethal dose of sodium pentobarbital. The subsequent post-fixation and clearing steps differed depending on the clearing and imaging method and are described below.

The 3-mm coronal brain sections were cut manually after the post-fixation step and a PBS wash using acrylic rat brain matrix (#BS-A 6000C, Braintree Scientific Inc.) and blades. Next the slices were processed accordingly per protocol.

### Confocal Imaging

For confocal imaging brains were cut on vibratome (VT100C, Leica) into 40 μm sections and processed for immunohistochemistry. Immunostaining was performed on free floating slices. GFP was detected using rabbit primary antiGFP antibody (#MB598, MBL) followed by incubation with secondary anti-rabbit antibody conjugated with AlexaFluor488 (#A-11034, ThermoFisher Scientific). NeuN mouse primary antibody (#MAB377, Merck Millipore) and anti-mouse antibody conjugated with AlexaFluore594 (#A-21201, ThermoFisher Scientific) were used to detect neuronal cells. Cell nuclei were counterstained with DAPI.

Confocal imaging was performed using LSM780 microscope (Zeiss). High-quality digital images were acquired using tiling mode and 10X objective. A large-scale brain mosaics were composed by stitching high resolution Z-stacks (tiles) that covered the whole brain. Next the specimen were imaged with 40X objective to obtain accurate details such as dendritic spines and contrast rich images.

### PACT Clearing

In case of PACT clearing, animals were perfused with ice cold PBS and 4% paraformaldehyde (PFA)/hydrogel solution as per procedure by Chung^5^ and then kept in hydrogel solution for one additional week in 4 °C. After 1 week of postfixation, brains were degassed and polymerized in 37 °C for 3 hours. The excess of polymerized hydrogel was gently removed and brains were subjected to *passive clearing* as per Tomer^16^. Brains were cleared for 4–8 weeks using clearing solution (borate-buffered 4% SDS) with gentle shaking in 37 °C. Clearing solution was exchanged every 24 hours for first 3 days and every week for consecutive weeks. After clearing, to remove remaining SDS brains were washed for 24 hours in PBS-T (PBS with 0.1% (vol/vol) Triton-X 100) in 37 °C. To match the refractive index of the tissue, brains were next kept in RapiClear© 1.47 Solution (#RC147, SunJin Lab) until sufficient transparency was reached. Due to prominent fragility of tissue following polymerization PACT was performed only on the whole brains.

Light-sheet imaging of PACT-cleared samples was performed using mineral oil (Sigma M8410, RI = 1.467) as the mounting solution (results not shown).

### CUBIC Clearing

Anaesthetized animals were transcardially perfused with a room temperature solution (RT) of phosphate buffered saline (PBS) followed by 4% paraformaldehyde (PFA) in PBS. Afterwards, the brains were removed and post-fixed at 4 °C in 4% PFA overnight followed by 2 washes in PBS at RT (each 30 minutes). Next, the tissue (whole brains and 3-mm slices) were processed according to the CUBIC protocol as described by Susaki^6^. Briefly, brains were incubated in CUBIC Reagent 1 (25 wt% urea (#U5378, Sigma), 25 wt% N,N,N’,N’-tetra-kis(2-hydroxypropyl) ethylenedi- amine (#122262, Sigma), and 15 wt% Triton X-100 (#X-100, Sigma)), for 2 weeks (whole brains) or 6 days (3-mm slices) in 37 °C. Reagent was exchanged every day. Next for further clearing, samples were incubated in CUBIC Reagent 2 (50 wt% sucrose, 25 wt% urea, 10 wt% 2,20,20′-nitrilotriethanol (#90279, Sigma), and 0.1% (vol/vol) Triton X-100 for 1 week (whole brain) or 2 days in RT.

Light-sheet imaging of CUBIC-cleared samples was performed using microscopy immersion oil (Cargille FF, RI 1.479) as the mounting solution. The samples can remain in oil for several days without loss of transparency. CUBIC Reagent 2 is not suitable as imaging medium for LSFM due to extremely high viscosity and tendency to crystallize below room temperature.

### FluoClearBABB clearing

Same as for CUBIC, the anaesthetized animals were transcardially perfused with a room temperature solution (RT) of phosphate buffered saline (PBS) followed by 4% paraformaldehyde (PFA) in PBS. Afterwards, the brains were removed and post-fixed at 4 °C in 4% PFA overnight followed by 2 washes in PBS at RT (each 30 minutes).

BABB tissue clearing was performed using the modified, fluorescent-protein preserving FluoClearBABB protocol^10^. Brains were post-fixated in 4% PFA at 4 °C for 24 hours, then washed two times in PBS. Dehydration of samples was performed in increasing tert-butanol (#360538, Sigma) concentrations (30%, 50%, 80%, 96%, 100%), each step for 24 hours, followed by 1 hour incubation in 100% hexane (#296090, Sigma). Clearing step included two days in BABB solution (benzyl alcohol (#13160, Sigma) and benzyl benzoate (#B6630, Sigma-1:2). BABB solution was exchanged after 24 hours. Triethylamine was added for pH adjustment to 9.5 value for alcohol and BABB solutions. Both dehydration and clearing procedures were performed in 30 °C.

Imaging was performed using the BABB clearing solution as the mounting medium in a 30-mm glass cuvette (#Z805866, Hellma) serving as imaging chamber, since the BABB solution is not compatible with plastic. The sample was mounted on a glass slide with cyanoacrylate glue (e.g. Superglue) which is not dissolved by BABB.

### Transmittance measurement

Transmittance was measured with a spectrophotometer (Hitachi U-2900). The slice was placed in a 10 mm cuvette filled with the mounting medium (BABB or Reagent 2 depending on the protocol).

### Light sheet microscopy setups

#### OpenSPIM for brains setup

The schematic diagram of the microscope is shown in Figure S1A. An argon ion laser (Lasos) is used for illumination. The 488-nm line is selected with a filter (Thorlabs FL488-10). The subsequent part of the setup is based on OpenSPIM^20^. First, the beam is expanded with a 5X magnification telescope (f = 25 mm and 125 mm) to a full width at half maximum (FWHM) of 3.6 mm so that the whole field of view of the detection objective is illuminated. Next, expanded beam is focused with a cylindrical lens (f = 75 mm). Its focal plane is imaged onto the back focal plane of the illumination objective (Nikon 4X Plan Fluorite, 0.13 NA) using a relay telescope consisting of 2 identical lenses (f = 50 mm). The objective is placed close to the window of the imaging chamber. All optical elements apart from the mirrors are placed on optical rails (Thorlabs RLA series) for easy alignment. The sample is mounted on a glass slide placed in a custom-designed holder attached to the arm of a 4D stage enabling XYZ translation and rotation around the vertical axis (Picard Industries). For CUBIC-cleared samples we designed our own sample chamber and 3D-printed it using ABS as material. 24 mm X 24 mm cover glasses attached with epoxy glue were used as windows. The sample was imaged using another Nikon 4X-PF objective and a Hamamatsu Orca-Flash4.0 camera. The water-dipping objectives from the original OpenSPIM setup have insufficient working distance for whole-brain imaging. Therefore our setup uses air objectives with long working distance (WD), which is the same approach as in the original ultramicroscope^2^ and other subsequently described setups^14^,^15^. WD of the Nikon 4X-PF objective is 17.2 mm in air, which corresponds to about 25 mm in cleared-tissue mounting media. The setup is controlled with the Micromanager software.

#### DIDV-SPIM setup

The schematic diagram of the microscope is shown in Figure S1B. CW lasers with wavelengths of 488 nm (Cobolt MLD, 150 mW) and 515 nm (TOPTICA iBeam Smart 515-S, 100 mW) are used for excitation. A flip mirror allows switching between the two lasers and a variable neutral density filter controls the laser power applied to the sample. Laser beams are expanded using a telescope system, composed of an objective lens (Edmund DIN 10, 10x, N.A. 0.25) and a plano-convex lens (Thorlabs LA1461-A, f = 250 mm), creating a flat-top Gaussian beam profile. Once the beam has been expanded, it passes through the rectangular slit (OWIS SP60), in order to adjust the illuminated area to the camera field of view. We use a 50/50 beam splitter (Thorlabs BSW10) to create double side illumination. Each of two illumination arms is composed of a 250 mm focal length cylindrical lens (Thorlabs LJ1267RM-A) mounted on a manual translational stage to control light sheet waist positioning. An additional mirror is mounted on a manual translational stage (OWIS VT30), allowing axial displacement of the light sheet, simplifying the alignment procedure. This is important taking into account that depending on the experiment, different chambers’ geometries, immersion media and detection paths are used which affects the light-sheet position. Samples are glued on half cut microscope slides mounted on a specially design sample holder. Scanning of the clarified brains is performed by translation of the sample with a motor (PI M-505.6DG) through a fixed light sheet plane. In order to image the whole brain different areas are acquired sequentially, controlled with two DC motorized stages (Thorlabs MTS50/M-Z8) in XY configuration, and afterwards stitched using Fiji plugins. Fluorescence generated at the sample can be collected in two different configurations. On one side a photography macro objective (NIKON Micro-NIKKOR 55 mm, f/2.8, NA 0.178) allows obtaining large FOV at resolution of 3.7 μm per pixel. On the opposite side, a microscope objective (Nikon Plan Apo 10x, NA 0.45, WD 4 mm) permits to collect higher resolution images of selected areas when needed. In both cases laser light is filtered out using a GFP filter (Chroma HQ515/30M-2P) and an achromatic doublet tube lens of 100 mm (Thorlabs AC254-100-A-ML and AC254-100-A-ML) creates an image on two different CCD cameras (Hamamatsu Orca-R2 and QImaging QCam, respectively). Due to space limitations, the high resolution arm camera is mounted vertically on a rail and cage system components are used to hold the objective lens. All the components of the setup are controlled using the free software Micromanager^28^ through the extended version of the plugin OpenSpinMicroscopy^29^,^30^.

### Image processing

Image processing was carried on in Fiji using the plugins Pairwise Stitching, Grid/Collection stitching^31^, and Variational Stationary Noise Remover^32^. 3D rendering was carried out using Imaris.

## Additional Information

**How to cite this article**: Stefaniuk, M. *et al.* Light-sheet microscopy imaging of a whole cleared rat brain with Thy1-GFP transgene. *Sci. Rep.* **6**, 28209; doi: 10.1038/srep28209 (2016).

## Supplementary Material

Supplementary Video 1

Supplementary Video 2

Supplementary Information
