# Supplementary material for: Light-sheet microscopy imaging of a whole cleared rat brain with Thy1-GFP transgene
Source: Sci Rep. 2016 Jun 17;6:28209. doi: 10.1038/srep28209 (PMC4911560; doi:10.1038/srep28209)
Supplement: Supplementary Information [file srep28209-s3.pdf]

Light-sheet microscopy imaging of a whole cleared rat brain with Thy1-GFP transgene

Marzena Stefaniuk[1#], Emilio J. Gualda[2#], Monika Pawlowska[1#], Diana Legutko[1], Paweł Matryba[1], Paulina Koza[1], Witold Konopka[1], Dorota Owczarek[1], Marcin Wawrzyniak[1], Pablo Loza-Alvarez[2]*, Leszek Kaczmarek[1,2]*

[1]Nencki Institute, Pasteura 3, 02-093 Warsaw, Poland

[2]Institut de Ciencies Fotoniques (ICFO), Barcelona Institute of Science and Technology, 08860, Castelldefels (Barcelona), Spain.

[#]Equal contribution

*Corresponding authors: l.kaczmarek@nencki.gov.pl, pablo.loza@icfo.es

Supplementary information:

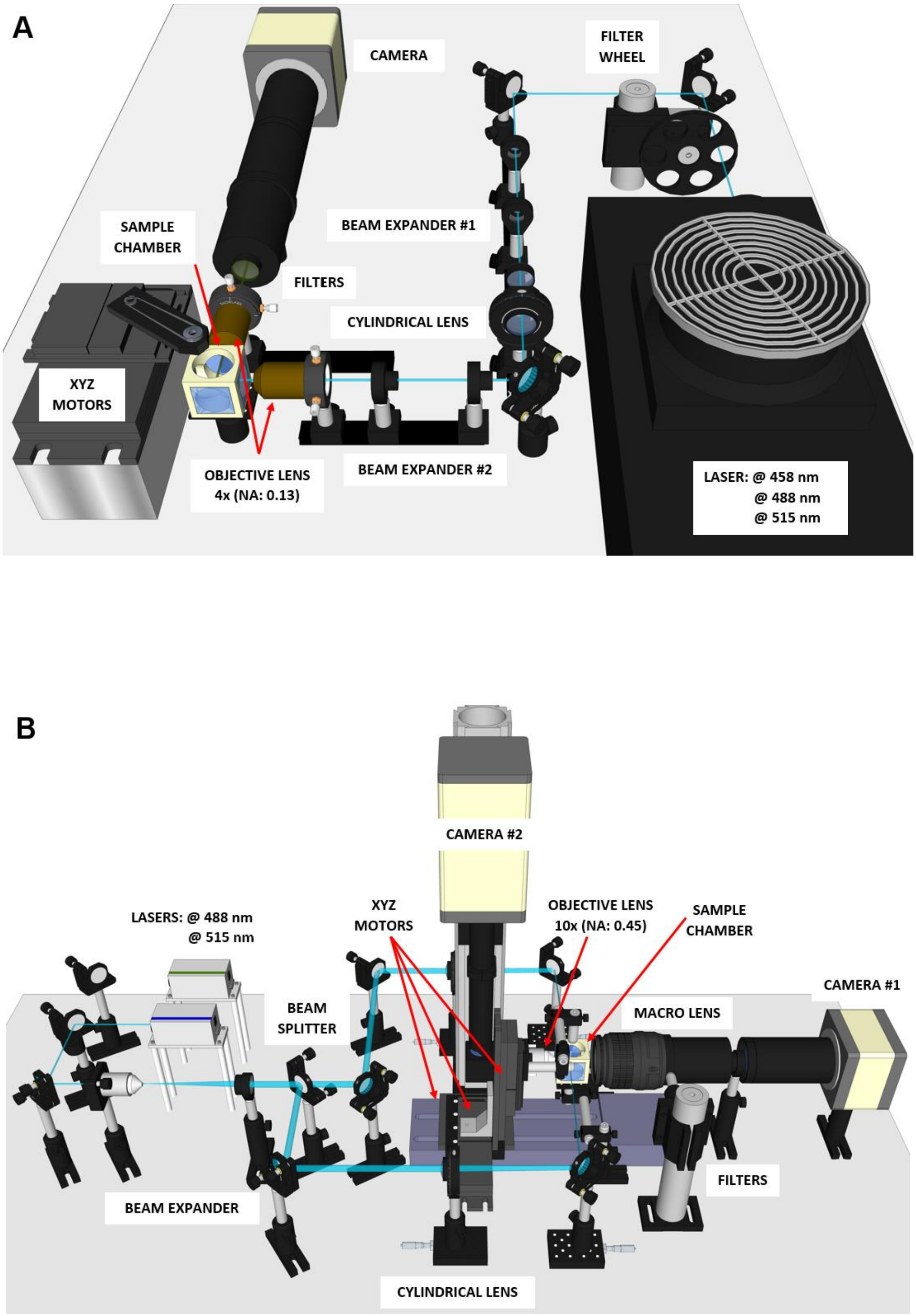

Figure S1 3D representation of LSFM setups used in this work. **(A)** OpenSPIM setup modified for brain imaging. **(B)** Dual-illumination-dual-view setup (DIDV). Blue lines indicate the laser beam path.

Supplementary Video 1 Z stack of the Thy1-GFP rat brain hemisphere cleared using FluoClearBABB technique, shown in **Figure 5**. The data set consists of eight stitched 3D stacks acquired using a macro objective and with double side illumination (four from each side).

Supplementary Video 2 Coronal view of the Thy1-GFP rat brain hemisphere consisting of orthogonal projections of the dataset displayed in Supplementary Video 1.
